# The introduction of advanced practice physiotherapy within Dutch primary care is a quest for possibilities, added value, and mutual trust: a qualitative study amongst advanced practice physiotherapists and general practitioners

**DOI:** 10.1186/s12913-022-07906-6

**Published:** 2022-04-21

**Authors:** Sylvia Pellekooren, Marianne Donker, Eddy Reijnders, Lamyae Boutalab, Raymond Ostelo, Maurits van Tulder, Annelies Pool-Goudzwaard

**Affiliations:** 1 Department of Health Sciences, Faculty of Science, Vrije Universiteit Amsterdam, Amsterdam Movement Sciences Research Institute, De Boelelaan 1105, 1081 HV Amsterdam, The Netherlands; 2 Department of Human Movement Sciences, Faculty of Behavioral & Movement Sciences, Vrije Universiteit, Amsterdam Movement Sciences Research Institute, Amsterdam, The Netherlands; 3 Huisartsopleiding VUmc, Amsterdam UMC, Location VUmc, Amsterdam, The Netherlands; 4 Department of Epidemiology and Data Science, Amsterdam Movement Sciences Research Institute, Amsterdam UMC, Location VUmc, Amsterdam, The Netherlands; 5 SOMT University of Physiotherapy, Amersfoort, The Netherlands

**Keywords:** Advanced practice physiotherapy, Extended scope specialist, Extended scope practitioners, Primary health care, Physiotherapy, Musculoskeletal complaints, General practitioner care

## Abstract

**Background:**

Despite the increased deployment and added value of Advanced Practitioner Physiotherapy (APP) in musculoskeletal care internationally, APP is not yet widely accepted within Dutch primary care. This may be due to specific constraints in the implementation of APP within the Dutch healthcare system. This study aimed to explore the experiences and perceptions of Advanced Practitioner Physiotherapists (APPs) and General Practitioners (GPs) with respect to implementing APP within Dutch primary care.

**Methods:**

This explorative and interpretive qualitative study included 12 APPs and 3 GPs who were in various stages of implementing an APP care model. Semi-structured interviews were conducted between January and March 2021. The topic list was based on existing literature, the personal input of researchers, and the Constellation Approach framework. Data were analysed using a thematic inductive approach.

**Results:**

Four main themes emerged from the data; 1) Both GPs’ trust in APP and a clear added value of APP are critical for starting implementation, 2) APPs need continuous support from GPs, 3) APPs believe that their position needs strengthening, and 4) Implementation of the APP model creates tension over ownership. These four themes highlight the perceived difficulties in gaining trust, lack of clarity over the added value of APP, ambiguity over APPs’ professional profile and positioning, a need on behalf of GPs to maintain authority, lack of reimbursement structure, and the struggle APPs face to strike a balance with current care.

**Conclusion:**

This study demonstrates that implementing an APP model of care is challenging, in part, because the deployment of APP does not sufficiently align with the core values of GPs, while GPs appear reluctant to hand over control of elements of patient care to APPs. APPs do not appear to have ownership over the implementation, given their strong dependence on the practice, values and needs of GPs.

**Trial registration:**

Ethical approval was obtained from the Medical Ethics Committee of VU University Medical Centre in Amsterdam; reference number 2020.17 . All participants were asked to provide written informed consent prior to participating in the study.

**Supplementary Information:**

The online version contains supplementary material available at 10.1186/s12913-022-07906-6.

## Introduction

Healthcare utilisation has steadily risen over the years and is expected to increase even further as a result of healthcare innovations and an aging population [1]. Similar to other healthcare systems in the world, the Dutch healthcare system faces the challenge of how to deal with the increased demand for care, which, in turn, increases the workload for healthcare workers. Dutch general practitioners (GPs) in particular, who are taking over tasks from secondary healthcare facilities while, simultaneously, seeing a decline in the number of their colleagues [2], have reported a considerable increase in their workload [3].

One way to reduce the workload of GPs is to deploy additional care givers, such as, for example, nurse practitioners and physician assistants, who have taken over the delivery of care for patients with chronic conditions like diabetes [4] and depression [5–7]. In light of these examples, there have been various initiatives across the globe to relieve GPs of having to care for the significant group of patients with musculoskeletal conditions seeking treatment, via the use of Advanced Practitioner Physiotherapists (APPs), who are also referred to as Extended Scope Practitioners (ESPs) [8, 9].

APPs operate at an advanced level of practice and provide care that is traditionally provided by other medical professionals, and are responsible for setting and communicating diagnoses, triaging for surgery or surgical opinions, ordering diagnostic imaging or laboratory tests, and prescribing/injecting medications [9, 10]. A recently published study showed that the deployment of APPs contributes to the accessibility of care with comparable health effects, diagnostic accuracy, and patient satisfaction [9]. In addition, the Advanced Practice Physiotherapy (APP) model of care has been shown to result in lower healthcare costs compared to usual care [11]. Based on these international findings, APP thus appears to be an appropriate alternative in treating patients with musculoskeletal conditions, which, in turn, may help reduce GPs’ workload.

In response to these international developments, APP was introduced in the Netherlands a few years ago via ESPs. This name was in accordance with extant literature at that time, which described the substitution of medical care by a physiotherapist as an ESP. However, the word practitioner was replaced by specialist to emphasise the difference between a physiotherapist qua practitioner, who treats patients via regular care, and a specialist who has more extensive tasks and responsibilities [8].

Despite promising results internationally, the deployment of APP within Dutch primary care has yet to garner wide acceptance. This may derive from barriers in the implementation of such innovations within the Dutch healthcare system, which is characterised by a demand-driven system with regulated competition and elements of both public and private insurance. All residents are entitled to a comprehensive health insurance package. This so-called basic health insurance package is compulsory, and the reimbursement structure is determined by the government. In addition to the compulsory insurance package, residents can also opt for supplementary insurance for forms of care that are not reimbursed by basic health insurance, such as, for example, physiotherapy. Within primary care in this healthcare system, there is a central role for the strongly positioned GP [12, 13], who not only functions as the gatekeeper for secondary care but also serves as the fixed first point of contact as all residents are registered with their own GP. Consequently, there is a long-term relationship between patients and GPs, allied with a strong focus on shared decision-making and high continuity of care [13].

Several qualitative studies [14–18] have explored the barriers and facilitators to the implementation of APP within secondary healthcare settings, concluding that knowledge, skills, availability of APP, motivation, and experience all have a large impact upon its successful implementation. Although these studies are undoubtedly of great value in terms of improving the implementation of APP, their outcomes are not necessarily applicable to the Dutch primary care setting. This is because these studies focused on the implementation of APP within secondary care facilities in other healthcare systems, and, as such, those barriers and facilitators that are specific to the Dutch primary care setting may not have been addressed. Therefore, this study aimed to explore the experiences and perceptions of Dutch APPs and GPs in implementing APP within Dutch primary care. Although the term Extended Scope Specialist  is used in the Netherlands, the acronym APP(s) is used throughout the manuscript, hereby following the term used in recent publications [8–11].

## Method

### Design

This was an explorative and interpretive qualitative study among Dutch APPs and GPs who were in various stages of implementing a musculoskeletal APP care model within a primary care setting. Semi-structured interviews were carried out between January and March 2021.

### Framework

The framework used to enrich the interview guide was the Constellation Approach [19], which was developed to analyse transitions in complex systems. The constellation approach assumes that complex societal systems, such as, for example, the Dutch healthcare system, consist of several subsystems, or so-called constellations. These constellations interact with, and adapt to, each other as well as their system’s environment. Each constellation comprises three elements, namely structure, culture, and practice. Structure refers to the physical, economic, financial, legal, organisational, and power structures that influence the behaviour of actors within a constellation. Practice concerns the actual actions that are undertaken within a constellation, such as the interactions between healthcare professionals and patients or between professionals and management. Culture pertains to the set of values, perceptions, and interpretations of actors within the constellation (e.g., patients, physicians, healthcare providers and insurance companies). While a constellation may change as a result of a variety of influences originating from within the organisation, it can also be demand- or supply-driven. When implementing APP in the Netherlands, the critical question is whether the healthcare structure, the beliefs of those parties involved, and the daily practice of musculoskeletal care are open to the introduction of APP.

### Participants

In order to achieve the maximum degree of variation and collect meaningful experiences and perceptions, participants were recruited via different channels. APPs were recruited through the researchers' existing network, which consisted of a group of approximately 30 APPs who were already known to SP as part of an observational pilot study. In parallel with this, participants were also recruited through both alumni and professional associations to include participants that were in other phases of implementation. These APPs were contacted via social media and through a call in the newsletters of the professional association. APPs were selected based on the stage of their implementation (e.g., start-up phase or established practice), personal characteristics (e.g., years of experience, region and attended training), and practice characteristics (e.g., self-employed or embedded in GP practice). GPs were recruited through the researchers' existing network and regional GP associations, and were approached by phone or email. On the whole, the willingness of GPs to participate was limited since these GPs were unfamiliar with APP, did not endorse it as a model of care, or indicated that they had no difficulties in providing care for patients with musculoskeletal complaints and were, therefore, not interested in the topic. As GPs proved to be a difficult group to recruit, they were selected based on convenience sampling. Twelve APPs and four GPs were included in the study, of which one GP subsequently decided not to participate due to their busy schedule.

### Data collection and data analysis

The topic list was based on the available knowledge from extant literature [14–18], before then being enriched with elements of the constellation approach (i.e., culture, structure, and practice characteristics) [19] and the personal input of the researchers (SP, ER, AP, RO, and MvT). SP’s personal input resulted in the inclusion of topics related to the APP perspective, and ER’s input led to the inclusion of topics focused on GPs’ perspective. Subsequently AP, RO and MvT checked the topic list for completeness and the neutrality of the questions. The final topic list included the following: reasons for starting a collaboration, extent of implementation and activities of APPs, training of APPs, awareness and need for APPs, support from the professional association, barriers and facilitators in the collaboration, alignment with the core values of Dutch general practice, and opportunities and future prospects for APPs. A full overview of the topic lists can be found in Additional file 1.

The interviews lasted around 60 min, with the exception of one interview that lasted thirty minutes, and were conducted via an online video call. The interviews were audio recorded and fieldnotes were taken. A pseudonymised verbatim transcription of the audio recordings was obtained. Summaries (i.e., member checks) were sent to the participants to provide them with the opportunity to comment and adjust the summary of their interview [20]. As part of this process, we stressed that the summary was the researcher’s interpretation of the interview and that any changes or additions were welcome. Five of the participants had some small remarks for clarification, which were accounted for during the further analysis. After reading their summary, some APPs expressed their disappointment and frustration toward the lengthy and cumbersome process when they became aware of the actual level of implementation. This, in turn, influenced the analysis and the subsequent development of themes.

Data were thematically analysed by means of an inductive approach [21]. Two researchers (SP and LB) familiarised themselves with the data by reading and rereading the transcripts, before subsequently independently selecting relevant fragments from three interviews by assigning open codes. These codes were then discussed and agreed upon with a third researcher (MD). After coding the first three interviews, a set of open codes were composed, which the remaining interviews were then coded with. Within this set, it was possible to add new codes. Any new codes were discussed within the research team and the set of codes were then adjusted accordingly, if necessary. Next, the codes were compared and grouped according to main- and subthemes. After interviewing nine APPs, no new themes were found from the APPs’ perspective. However, we did decide to conduct an additional three interviews with APPs to explore the GP perspective further, as we felt that the GP perspective was less reflected as a result of having only conducted three interviews with GPs. We found that in prior interviews with APPs, APPs had also put forward elements of the GP perspective, so we thought that additional interviews could help contribute to a better understanding of the GP perspective. However, no new themes emerged from these additional interviews. Valuable quotes were selected during the analysis and then discussed and interpreted among the researchers (SP, MD). All analyses were carried out in MAXQDA (version 2020).

### Ethical considerations

All participants provided their informed consent prior to participating in the study. Ethical approval was obtained from the Medical Ethics Committee of VU University Medical Centre in Amsterdam; reference number 2020.17.

## Results

The APPs who participated in the study differed in terms of their personal characteristics and specific working conditions, such as, for example, their forms of reimbursement and level of organisational embedment. Seven of the twelve APPs collaborated with a GP, of which two worked under the supervision of the GP and five worked independently. More details on the participants can be found in Table 1.

**Table 1:** Characteristics of the participants

Respondent	Gender	Age	Years of work experience as PT	Years after graduating APP	Currently practicing APP	APPs working under supervision	
APP 1^a^	Female	> 50	> 25	> 2	Yes	+ ^b^	
APP 2^a^	Female	> 50	> 30	> 2	No	+ ^b^	
APP 3^a^	Female	> 60	> 30	> 2	Yes	+	
APP 4^a^	Male	> 50	> 20	> 2	Yes	+	
APP 5	Female	> 35	> 10	< 2	Yes	- ^c^	
APP 6^a^	Female	> 45	> 20	< 2	Yes	- ^c^	
APP 7^a^	Male	> 55	> 35	> 6	Yes	+	
APP 8^a^	Female	> 40	> 20	> 2	Yes	+	
APP 9^a^	Female	> 40	> 15	> 4	No	Na	
APP 10^a^	Male	> 30	> 10	> 2	No	Na	
APP 11^a^	Male	> 40	> 15	< 2	No	Na	
APP 12^a^	Male	> 40	> 20	< 2	No	Na	
**Respondent**	**Gender**	**Age**	**Years of work experience**	**Practice composition**	**Number of patients registered to GP practice**	**Number of collaborating APPs**	**APPs working under supervision**
GP1^a^	Male	> 55	25	1 GP, 1 permanent alternate	2200	2	+
GP2^a^	Female	> 40	13	2 GP	2900	1	-
GP3^a^	Female	> 50	21	1 GP, 1 HIDHA, 1 HAIOS	3000	1	+

*APP* Advanced Practitioner Physiotherapy, *APPs* Advanced Practitioner Physiotherapists, *GP* General Practitioner, *PT* Physiotherapist, *HIDHA* GP employed by another GP, *HAIOS* GP in training

^a^Owner practice, ^b^Joint consultation, ^c^Independent consultation

Four main themes derived from the data; 1) Both GPs’ trust in APP and the clear added value of APP are critical for starting implementation, 2) APPs need continuous support from GPs, 3) APPs believe that their position needs strengthening, and 4) Implementation of the APP model creates tension over ownership. Details on the derived subthemes and axial codes can be found in the code tree, which is presented in Table 2.

**Table 2:** Code tree

Themes	Subthemes	Axial codes
Both GPs’ trust in APP and a clear added value of APP are critical for starting implementation	GPs need to trust APP	
	GPs doubt added value of APP	
APPs need continuous support from GPs	APPs need the full commitment of GPs to start	APPs cannot refer to secondary care on their own
		Limited availability of patient information
		Triaging patients lacks criteria
		APPs and GPs want to scale-up
		GPs and APPs struggle with who is in charge of the care pathway
	APPs require support from GPs while they build-up their self-confidence	Insecurity during delivery of care
		Insecurity during team interactions
		More work experience increases their self confidence
	Establishment of proper reimbursement is crucial	
APPs believe that their position needs strengthening	GPs want to retain their authority and control	Competencies and attainment levels are poorly crystalised
		Different preferences for type of employment and final responsibility
		APPs experienced tension between GPs’ standards and their working methods
	More guidance from the professional association is desirable	APPs want more backing from trade organisation
		Trade organisation needs to be a driving force towards stakeholders
	APPs found limited added value in the training they attended	Work experience influences the added value of the training
		Curriculum needs more in-depth and practical training
Implementation of the APP model creates tension over ownership	No place for APP among physiotherapy yet	Gaining trust amongst physiotherapists with whom they need to collaborate
		Controversy over the positioning of APPs
	Finding the balance between taking over GP care and safeguarding core values	Deployment of APP jeopardises patient-centred care
		Ensuring the independent delivery of care appears to be an unfeasible ideal
		GPs must be able to maintain the delivery of general medical care at a qualified level
		APPs and GPs need to develop a common language

### Both GPs’ trust in APP and a clear added value of APP are critical for starting implementation

#### GPs need to trust APP

All the APPs indicated that having a long-term relationship with a GP is a prerequisite for introducing an APP model of care. GPs need to trust in both the competencies and motivations of APPs in order to develop confidence in the collaboration and eventually hand over care delivery to APPs. This trust can be built by working together. Those APPs that lacked such a pre-existing relationship experienced difficulties in connecting with GPs, gaining their trust, and introducing an APP model of care without calling into question GPs’ competences.

*The most important factor is trust. Trust that those who are doing the project, APP X and APP Y, are competent in the matter. That they are also prepared to behave in this way, and not say, this is a disguised way of bringing in more clients at the end of the day, so that is the most important thing, I think. [GP 1]*

#### GPs doubt added value of APP

Many GPs are still unfamiliar with APP and what the profession precisely entails. GPs find it difficult to see both the added value of APP over specialised physiotherapy and how an APP model of care would improve their current practice.

*Because of course there are so many different therapists with all kinds of functions. It has to be very clear what exactly the added value is for us to refer a patient to an APP instead of a 'regular' physio. [GP 2]*

In addition, the GPs indicated that it remains unclear what an APP model of care offers them personally and professionally, whether it be in terms of time savings or better quality of care. All GPs reported that one-off assessments by APPs would undoubtedly contribute to greater musculoskeletal expertise within their GP practice, while one GP mentioned that potentially reducing their own workload gave it added value. However, two of the three GPs interviewed indicated that heavy workloads were not primarily caused by patients with musculoskeletal problems, but by the relocation of care from secondary care settings, such as, for example, mental healthcare facilities and care for the elderly.

*And the problems with the elderly are just very heavy, when you have so many elderly. I have a lot of elderly people, and they all live at home, and I have a lot of demented people, and there is little home care. It is a familiar story. Not enough places, they cannot be admitted, or do not want to be admitted. That is what takes up most of my time. That will continue to be my practice. So that is where I need the most support actually. [GP 3]*

### APPs need continuous support from GPs

#### APPs need the full commitment of GPs to start

The vast majority of APPs indicated that the start-up stage of an APP model of care is a long process that involves many steps, especially for APPs that are not embedded in the GP practice, who also must deal with legislative issues like the General Data Protection Regulation and doctor-patient confidentiality. The APPs indicated that receiving support from GPs is essential for referring to secondary care, eliciting enough patient information for setting out the care pathway properly, and for setting up referral streams. However, some APPs experienced that GPs tend to be less committed in implementing an APP model of care since the interest mainly lies with APPs. Although embedding APP within GPs’ practice can overcome some of these aforementioned hurdles, it is not attractive to all GPs because it means taking on more staff.

*And I can only speak for my own GPs, something I've discussed a lot over the last year, GPs don't want to grow in the size of their practices either, they're not waiting for 30 practice support staff. The role that we have now is actually quite fine, nice, I don't have anything to do with you, I don't have to take care of you when you're sick, you take care of it there, we take care of it here, that's what these GPs like very much. And my GPs are not waiting for APP to come in as well. [APP 4]*

#### APPs require support from GPs while they build-up their self-confidence

All practicing APPs sometimes feel insecure and vulnerable over having primary responsibility for patients’ wellbeing, especially when their complaints may not appear to be related to the musculoskeletal domain. Having consultations with fellow APPs or the authorising GP helps to reduce this uncertainty. Practicing APPs expressed feeling uncertainty when reporting to GPs and felt that they were not allowed to make mistakes in the initial stage where they still had to prove themselves. Their self-confidence would grow by receiving positive feedback from GPs and gaining more work experience.

*It would be a death blow of course, everyone makes mistakes, but it would mean the end of everything if we had a lot of misdiagnoses in the initial phase. Then, immediately, seeds of doubt are sown, and of course, we cannot have that. [APP 4]*

#### APPs needs practical support from multiple GPs to carry out their practice

Some APPs and GPs indicated that a uniform way of working and communication are paramount for both ensuring high-quality care and for carrying out joint consultations to this end. All APPs and GPs preferred a workplace within a health centre where several GPs work, because APPs are then embedded in the GP practice and short lines of communication are established. However, this is difficult to realise in practice due to the lack of working space within most health centres. A few APPs stated that working out of one’s own physiotherapy practice is attractive, as this increases the referral of patients for physiotherapy treatment, and, as such, one’s income. The GPs indicated that working out of one’s own physiotherapy practice is not desirable, as the independence of care and the role of APPs then comes into question. All participants saw the added value of scaling up the team, as far as this ensures continuity, independence, and quality of care. All GPs indicated that they struggle with referring a sufficient number of patients and are uncertain over which APP they should be contracting. A few APPs mentioned that it is difficult to scale up due to both the insufficient number of trained APPs in their work area and the competitive attitude of other APPs.

*I think that in our case she [APP] should actually work for several practices, because one practice – even though I have a large practice – one should have more opportunities available. You always have people who think, I would rather go to the GP because then I will see the doctor again, too. Or imagine, you have already been through a lot with a patient and then the patient prefers the GP. Not that it is necessarily better in terms of content, but because the GP is a trusted figure. [GP 3]*

#### Establishment of proper reimbursement is crucial

All APPs and GPs indicated that the lack of an appropriate financing structure is a major barrier for APPs, GPs, and patients. Although reimbursement is possible through the health insurer’s innovation fund, GPs are either not able or are unwilling to utilise this. As patients are used to GP care being reimbursed from their public health insurance, GPs would not only have to convince patients of the added value of an APP over a physiotherapist, but also inform them about the additional costs. These costs would then either be paid out of patients’ own pockets or at the expense of the number of physiotherapy treatments covered by their supplementary insurance. Some GPs indicated that they perceive this restricted accessibility of care based on a patient’s financial position as unpleasant and/or unethical.

*GPs were not really keen on using funds from the innovation fund of the health insurers for this purpose. Many GPs had also just made additional investments in physician’s assistants. So that was an issue. Also, because we have another group of GPs here, some of whom think that extended scope is unnecessary. [APP 10]*

### APPs believe that their position needs strengthening

#### GPs want to retain their authority and control

The APP competency profile developed by the Dutch professional association for APP is unknown to many APPs and leaves room for differentiation in the function of APP. All of the participating APPs had different views on competencies, end terms, tasks, patient population, and their position in the care pathway. Some APPs indicated that this flexibility in their profile leads to ambiguity and confusion amongst GPs and patients. There is no consensus yet amongst both APPs and GPs over the establishment of employment of APPs within the GP practice and if APPs should work according to GP professional standards. Some APPs who do set out the care pathway themselves indicate that, despite agreements made, they sometimes have trouble staying in charge of the treatment plan, as in practice their role is also influenced by old behavioural patterns of patients and GPs. In addition, most APPs argued that their role as APP seems not only to be determined by the professional profile but also by the extent to which APP is allowed to work next to the GP by the GP. The APPs also indicated that they are cautious in taking over too much care at the one time and proceed step by step to avoid resistance from the GP.

*Initially that would not matter to me. I think that we should say that, as a goal, it will eventually be fully under APP own authority. Certainly, to get the GPs on board I think that you must first do this under the GP’s authority, until they themselves conclude, no, you can do this on your own just fine, and I don’t need to be behind this, like some version of extension of care. So, I think that this must be introduced step by step. In particular if you also notice that they [GPs] are going to get up in arms, then you should introduce that very slowly. And prove yourself first. You must. [APP 6]*

Although the APPs indicated that they are willing to temporally work under GP authority, two GPs stressed that they have no intention of handing over full authority. Rather, they stated that they will either opt for joint consultation or deploy APPs under supervision and set out the care pathway themselves, thereby retaining control.

*I should like it to be under my supervision because I think that in this way I can offer an extra service to my patients, a broader selection of diagnostic skills and I do not throw this [treatment responsibility] out. So, for as far as this goes, I want them [patients] to go to it [APP], and then they often return to me, and we discuss what the proposed treatment plan is. In this way I do not let go of them. [GP1]*

#### More guidance from the professional association is desirable

Almost all APPs stated that they missed the support of a professional association when starting their APP practice. That is to say, they missed having a platform to fall back on and get more guidance, such as, for example, a concrete plan of action, standard documentation, and advice on how to communicate with GPs, which was needed but not yet available. Virtually all the APPs felt that the professional association is not sufficiently visible to the various stakeholders, while developments within the professional association take a long time. All APPs indicated that the implementation of APP would benefit from a decisive board that is actively engaged in creating support amongst stakeholders. The lack of direction from the professional association leads to many individual initiatives, loss of control over this growing profession, and differences in the interpretation of the role and working method of APPs.

*I understand that as well, because it is a new association and must be built from the ground up. Furthermore, it is not their main task, they also have of course their own jobs to do. But certainly, for this project, things [documentation] have been agreed upon and were to have been sent in, but this has not happened, which is a pity, because as a pioneer, you really need support. And that is not happening. Or at any rate, too little. [APP 2]*

#### APPs found limited added value in the training they attended

The vast majority of the APPs interviewed said that the training they had undergone contributed little to the knowledge and skills they had already acquired in either their work as manual or sport physiotherapists or in their previous master’s degree courses. Some APPs indicated that, compared to other countries, the scope of the training was too limited, and that practical education under the supervision of a doctor was lacking.

*This is fine for a few weeks, going a bit deeper into things, but does not compare with the role they play abroad, nor the training they receive for this…. They have had a completely different training in this, and this I think, is what is keeping us from getting any further with this APP story in the Netherlands. [APP 9].*

### Implementation of the APP model creates tension over ownership

#### No place for APP among physiotherapy yet

All the APPs indicated that building a collaborative network with physiotherapists in their region costs them lots of time and effort, as the concept of APP is still relatively unknown. Feelings of anxiety over losing patients as well as unfair competition amongst physiotherapists both contribute to the slow acceptance of APP, despite the efforts of APPs themselves to stress that it is not their intention to treat patients themselves. Some APPs reported that with the current reimbursement APP acts as a competitor to physiotherapists, which has a deleterious impact upon their cooperation.

*How do I notice this happening? Not providing information, not sharing patients, getting angry with you the moment you see a patient and call about it, or do a report, or have an other idea. If you want to set up a project about APP care, and you go to a big player in the neighbourhood who also has a similar plan, something broader, and you say, well, let us join forces, then it is all impossible. No, it is all too sensitive, too much me, me, me…. This leads to extremely unpleasant conversations. [APP 1]*

Some APPs mentioned that combining the APP care model with direct access physiotherapy results in APP functioning as an additional gatekeeper along with the GP. This may be used as a unique selling point to expand one’s own physiotherapy practice and make more money, which, in turn, leads to feelings of unfair competition and resistance towards APP. One APP stated that there are ongoing discussions both in the field of work and at the management level who can be an APP and who cannot. Some APPs said that they had experienced that some physiotherapists present themselves as APPs without undergoing the proper training. Indeed, one APP even mentioned that the Royal Dutch Society for Physical Therapy (KNGF) agrees that at least in principle, every physiotherapist can carry out APP.

*The other one, practice X, just wants to scale-up. And they also want to be a part of it [setting up an APP practice in the region], but then it is no longer about the content. The worst thing I found, was that nobody has done training in APP, but they pretend to be on top of it... I think the Society, that is the regional representative of KNGF, believes that every physiotherapist should be able to be an APP. I do not agree with him at all. Manual therapy and sport physiotherapy may think so, but the KNGF has a completely different opinion. At least in our region, the KNGF simply airs this. This is already a difficult matter. [APP 3]*

#### Finding the balance between taking over GP care and safeguarding core values

All the GPs indicated that collaborating with APPs may jeopardise patients’ interests, due to a restricted choice of care provider and further fragmentation of care. All APPs and GPs endorsed that APP should operate as an independent point of care and emphasised that one should not position APP as part of the business model of one’s own physiotherapy practice. However, the APPs indicated that this independence is difficult to realise as both APP care and physiotherapy care are typically provided alongside each other, due to the limited number of patients, lack of workplace at healthcare centres, and poor understanding of APP services by patients. Most APPs stressed that providing independent care still has a long way to go and may in fact not be feasible, especially for those APPs that are affiliated with large physiotherapy practices that provide a wide variety of in-house treatment options.

*On the other hand, I discussed this [lack of independent care delivery], with fellow physios already during my training, and they all say, are you crazy, everyone works that way within primary care. And they all pass the buck to each other. So, I let it rest for a while. They are right, I think the same way, but that is partly a hypocritical remark for everyone. So, then everyone needs to put his own house in order, and then we can all be morally justified. But to be honest, because I am quite a moralist, if I let go of that, I think it is going to be a difficult issue. I agree, I totally agree, I think that is the way it should be, in the ideal world, but I think we are a long way from that. [APP 11]*

All the GPs mentioned that in order to provide proper general medical care, gaining and maintaining experience with musculoskeletal complaints is absolutely essential. Some APPs and GPs indicated that not all GPs are willing to hand over patients with musculoskeletal complaints due to their personal interest in this population and/or beliefs about the content of their profession and Dutch GP core values. Indeed, two out of three of the GPs interviewed felt that APPs still have to grow into the culture of the GP practice and find a way to connect with the core values. Some APPs noted that connecting with the GPs and relating to the mutual dynamics of GPs can be difficult due to other perspectives on the quality of care.

*Totally different, if you think it might be a good idea to involve a secondary care orthopaedic, then the GP says, oh, no, you mustn't, because that is seen as primary care in disguise. So, you definitely should not do that! You are just not aware of all these strategically sensitive things. And you think you have a great product, and the GP thinks, how so? I do not need you at all. So how are you going to connect with them? [APP 1]*

## Discussion

This study explored the experiences and perceptions of APPs and GPs towards both the implementation and deployment of APP within Dutch primary care and found that it is difficult for APPs to carve out a place for themselves within the healthcare landscape.

Within the present study, four themes emerged from the data through which APPs and GPs’ experiences of APP deployment and implementation can be understood. The first theme sheds lights on the fact that the success of APP depends on both the trust of the GP and whether they perceive it as having clear added value in comparison to the usual care. The second theme underscores that the support of GPs is essential for APPs, as far as it helps to, amongst other things, get different referral flows going. The GP also plays an important role in terms of building the self-confidence of APPs, in creating uniformity within patient care, and in terms of helping to bring about a team that works under one roof. The lack of funding for APP raises concerns over the deployment of APP among APPs, GPs, and physiotherapists. The third theme points towards the fact that the position of APPs needs strengthening. Indeed, the professional profile of APP is something that proved to be unclear to both GPs and APPs themselves. In the absence of a uniformed way of working, everyone is still searching, which, in turn, results in diversification. GPs’ reluctance to hand over control also profoundly impacts on the role of APPs. Amongst APPs, there is a need for better positioning, support, and profiling from the professional association as well as for training which includes more depth and practical education. The fourth theme pertains to both the tension that persists around ownership of patients with musculoskeletal complaints and the competition between APPs and physiotherapists. This is compounded by a lack of adequate funding and the ability to generate patient flow for the physical therapy practice to which APPs are affiliated. Moreover, the APP model seems to insufficiently adhere to GPs’ core values.

### Comparison with literature

Many of the themes identified are in accordance with earlier publications on APP, such as the role of trust and need for acceptance by doctors [14, 16], recognition of the added value by doctors [14, 16, 17] and the establishment of an appropriate financing structure [14, 17]. The present study shows that many of these previously identified factors, such as physician trust and demonstrating clear added value to stakeholders and the financing structure, have hitherto not been sufficiently realised to facilitate the implementation of APP within Dutch healthcare. The most important barrier, however, appears to be GPs’ reluctance to hand over authority and control. This appears to stem from specific characteristics of Dutch general practice, such as long-term doctor-patient relationships and GPs’ strongly held core values, but also derives from the traditional authority that GPs have over physiotherapists as a result of differences in educational level, which persists because of the lack of sufficient training and entrusted professional activities.

Introduction and support from within the organization have been described as helpful in studies of APP embedded in secondary or tertiary care [14, 15, 17, 18]. Such support is lacking in the implementation of APP in the Netherlands, and individual APPs working independently in primary care must build a partnership without any support.

A number of studies have shown that the availability of training at an appropriate level is critically important [14–16]. Our study shows that, according to the experiences of the APPs, both the form and scope of the current education is not in line with the demands of the professional field and, moreover, is not sufficiently different from their prior training and thus lacks added value for them. In addition, individual APPs are currently responsible for organising their own practical training in the field. It is unclear to what extent this is feasible for APPs given the limited scale of most of their collaborations, where guidance often has to be provided by an individual GP, while gaining practical experience is dependent on the limited number of patients registered with this GP.

Furthermore, a number of studies have shown that a clear delineation of the role of APPs and greater standardisation of working procedures is important [19, 20, 22]. A recent qualitative study examining the goals, roles and tasks of APPs in the Netherlands revealed that the participants found it difficult to state clear goals for APPs and that there is no consensus concerning the positioning of APPs [22]. A study on how best to shape the interprofessional collaboration between GPs and established healthcare professionals [23] showed that these collaborations do not always go well and that it is crucial to establish a shared vision and clarity over work structure, procedure, and role distribution. Awareness of each other's context and expectations was also found to play a key role. According to the APPs and GPs who took part in this study, a clearly defined role and standardisation of process and working methods of APPs has yet to be realised. This makes it incredibly difficult to develop the partnership between APPs and GPs.

Amongst GPs, there is a need to improve the already existing collaboration with physiotherapists to ensure the increasingly complex care of patients with musculoskeletal complaints [23]. Within current Dutch primary care, around half of all GPs already have an existing collaboration with a physiotherapist [24], while a large proportion of patients with musculoskeletal complaints visit a physiotherapist via Direct Access Physiotherapy [25]. In this context, the question is whether there is a need therefore for a new type of care provider, such as APPs, or whether there is a need to revise the existing collaborations with physiotherapists, by improving the level of communication and having one-off diagnostic consultations.

In other countries, such as Australia and the United Kingdom, APP has emerged in response to urgent demand from physicians [14, 16]. Here, involved stakeholders have felt sufficient urgency to change and, moreover, physicians have endorsed the need for the use of APPs [14–16]. Within the present study, there was no such urgency and need expressed by GPs. This might relate to differences in the organisation of healthcare systems, not to mention the good accessibility and continuity of Dutch GP care. It has also been found that when APP is not initiated by physicians themselves, then its implementation is altogether more difficult and dependent on goodwill [16]. This also appears to be the case with the implementation of APP in the Netherlands.

It remains to be seen to what extent APP fits within the Dutch College of General Practitioners future vision [26] in which the GP, as the first point of contact, maintains an overview of medical care and determines, together with the patient, what care is necessary and appropriate. The Dutch General Practitioners Association has recommended that, when entering a partnership with a new care provider, GPs must determine, before doing so, to what extent the core values and core tasks are to be guaranteed [27–29]. Moreover, GPs are advised to assess if the collaboration with this new care provider corresponds to their own preferences, ambitions, and vision of GP care [27–29]. In addition, a study amongst patients of Dutch GPs showed that patients’ wishes regarding healthcare providers should be considered in ever-increasing collaborations with the GP practice [30]. At present, it is not feasible for APPs to adequately align with the key conditions that GPs want to see fulfilled before they are willing to change their practices, while it remains unclear to what extent patients' wishes are being heeded in the implementation and deployment of APP.

The importance of connecting to core values was also highlighted in a study evaluating barriers to the implementation of the Dutch General Practitioners Association treatment standards [31]. This study demonstrated that, despite the positive attitude of GPs towards the implementation of these standards, GPs only follow the standards when they are in line with the core value of patient-centred care. This makes it clear that, even with an improved positioning of APP, connecting to the core value of person-centred care is decisive in successfully implementing APP. There seems to be a lack of vision regarding under what conditions this can be met, which, in turn, makes it difficult for individual APPs to connect with GPs.

### Strengths and limitations

One of the strengths of this study is its credibility [32]. The starting point was an extensive literature review, which subsequently formed the basis of the interview guide. Multiple researchers collaborated on this study, and during the analysis, two researchers coded independently of each other, and subsequently the codes and themes were extensively coordinated and discussed within the research team. In addition, the full scope of the use and implementation of APP was examined by using concepts from the constellation approach as sensitising concepts in developing the interview guide. Moreover, all the participants were sent a member check after the interview and their responses were included in the analysis. Another strength concerns the conformability [32] of the results, as a large team from different backgrounds worked on the study. Moreover, a good audit trial was carried out, during which the selection process around the analysis was recorded and explicit attention was paid to the views and thought processes of each individual team member. This was an important aspect as one individual researcher (SP) is a physiotherapist and was involved in conducting an observational pilot study that evaluated the APP model of care and, as such, was more familiar with the perspective of APPs. The presence of possible disconfirmatory cases was discussed within the research team, but although there was diversification amongst the participants, no disconfirmatory cases were identified. The findings were in line with other studies examining the implementation of APP models of care. The transferability [32] of the findings is unclear. Despite there being similar findings in extant literature on implementation level, comparison with international literature is difficult given the specific Dutch context. Although we used maximum variation sampling, we were compelled to recruit GPs through convenience sampling given the limited number of GPs who were willing to participate, which meant that we failed to include GPs who were not open to implementing the APP model. This probably hinders the transferability of our findings, as far as we may have missed aspects of the GP perspective. However, gaining trust in APP, the need for a clear added value, reluctance to hand over control, and strongly held core values was expressed by all the participating GPs. There may also be shortcomings in the dependability [32] of the findings. Although we collected data until no new themes derived and flexible analysis took place, data collection and data analysis were not a wholly iterative process. In addition, there is a possibility that some of the participants may have felt less free to express themselves during the interview, out of concern that they may have, despite being anonymised, been recognised by colleagues and stakeholders based on their specific characteristics.

## Conclusion

The results of this study show that implementing an APP model of care is challenging within the Dutch healthcare system. The deployment of APP does not sufficiently align with the core values of GPs, and GPs appear to be reluctant to hand over some control over patient care to APPs. Therefore, APPs do not appear to have ownership over the implementation, given their strong dependence on the practice, values and needs of GPs.

## Supplementary Information

**Additional file 1.**

## Data Availability

The datasets generated and/or analysed during the current study are not publicly available as no informed consent was obtained for this but are available from the corresponding author on reasonable request.

## Abbreviations

APP: Advanced Practice Physiotherapy
APPs: Advanced Practitioners in Physiotherapy
GP: General Practitioner
KNGF: Koninklijk Nederlands Genootschap voor Fysiotherapie (Royal Dutch Society for Physical Therapy)
