# Supplementary material for: The introduction of advanced practice physiotherapy within Dutch primary care is a quest for possibilities, added value, and mutual trust: a qualitative study amongst advanced practice physiotherapists and general practitioners
Source: BMC Health Serv Res. 2022 Apr 21;22:529. doi: 10.1186/s12913-022-07906-6 (PMC9026935; doi:10.1186/s12913-022-07906-6)
Supplement: Supplementary file 1 — Additional file 1. [file 12913_2022_7906_MOESM1_ESM.docx]

**ADDITIONAL FILE 1. TOPIC LISTS**

**Topic list General Practitioners**

Introduction question:

- What factors played a part in your decision to collaborate with an APP?

Topic 1: Collaboration with APP

- What positive aspects do you experience in the collaboration with an APP?
- What can be improved?

Topic 2: Tasks and responsibilities APP

- Would it matter to you if an APP performs tasks independently (task substitution) or under your supervision (task delegation)?

Topic 3: Core values GP

- To what extent do you think an APP is able to conform to GP core values? How important is this to you?
- How do you feel about APP taking over a part of GPs gatekeeper function?
- To what extent do you think an APP can provide independent care?

Topic 4: Workload and work pressure.

- To what extend do you experience workload caused by patients with musculoskeletal complaints?
- What impact has the deployment of an APP on your workload?
- To what extend do you experience work pressure caused by patients with musculoskeletal complaints?
- What impact has the deployment of an APP on your work pressure?

Topic 5: Health care utilisation and patients’ needs

- What effect do you think the deployment of APP will have on the referral rate of patients with musculoskeletal complaints?
- What effect do you think that the deployment of an APP will have on health care utilisation by patients with musculoskeletal problems?
- To what extend do you think patients feel the need for an APP?

Topic 6: Future

- How do you see the future of APP within general practice?
- What factors do you think will influence the implementation of APP in general practice?
- Would you consider entering a long-term collaboration with an APP?

**Topic list APP with a collaboration**

Introduction question:

- To what extend are you currently working as an APP?

Topic 1: Role and tasks APP

- Which patients are seen by you in the role as APP?
- At what place in the care pathway are you deployed?
- What tasks do you perform as an APP?
- Do you perform these tasks independently (task delegation) or under the responsibility of the GP (task delegation)?
- Does it matter to you whether you are working under task substitution or under task delegation?

Topic 2: Training, skills, and core values general practice

- How did the training prepared you to work as an APP?
- Do you plan to attend further training?
- For what tasks do you feel you competent?
- For what tasks do you not feel competent at this moment?
- To what extent do you feel you are able to provide independent care? How important do you think that is?

Topic 3: Implementation

- How did you experience the start-up of your APP practice?
- What was difficult?
- What was helpful?
- What practical aspects play a role in working as an APP?
- To what extend did you feel supported by the professional association to work as an APP?
- To what extend did you feel supported from GPs?

Topic 4: Awareness and patients’ needs

- To what extent do you expect that other healthcare providers are familiar with APP?
- How does this affect your work as an APP?
- To what extent do you expect patients to be familiar with APP?
- To what extend do you think patients feel the need for an APP?

Topic 5: Collaboration with GP

- What positive aspects have you experienced during your collaboration with a GP?
- What aspects have you experienced as difficult during your collaboration with a GP?

Topic 6: Future

- To what extend do you think that an APP care model seem to be a promising initiative within general practice?
- What factors do you think will influence the implementation of APP in general practice?

**Topic list APP without collaboration with a GP**

Introduction question:

- To what extend are you currently working as an APP?

Topic 1: Role and tasks APP

- Which patients could be assessed by an APP in primary care?
- At which place in the care pathway should an APP be deployed?
- Which tasks do you think are suitable for an APP?
- To what extent would it matter to you if you performed these tasks independently (task substitution) or under supervision (task delegation)?

Topic 2: Training, skills, and core values general practice

- How did the training prepared you to work as an APP?
- Do you plan to attend further training?
- For what tasks do you feel you competent?
- For what tasks do you not feel competent at this moment?
- To what extent do you feel you are able to provide independent care? How important do you think that is?

Topic 3: Implementation

- What aspects played a role in trying to set up an APP practice?
- To what extend did you feel supported by the professional association to work as an APP?
- To what extend did you feel supported from GPs?

Topic 4: Workload and workload

- To what extend does APP affect the workload of GPs?
- What influence do you think that the deployment of an APP has on the GP's work pressure?
- What do you think that the added value of APP is compared to other initiatives to address GPs’ workload?

Topic 5: Health care utilisation and patients’ needs

- What effect do you think the deployment of APP will have on the referral rate of patients with musculoskeletal complaints?
- What effect do you think that the deployment of an APP will have on health care utilisation by patients with musculoskeletal problems?
- To what extend do you think patients feel the need for an APP?

Topic 6: Future

- What opportunities do you see for APP within general practice?
- What factors do you think will influence the implementation of the APP in general practice?
- In what timeframe do you expect to start as an APP yourself?
- Where does this depend on?
